# Greetings from the Editor-in-Chief: Moran Ki

**DOI:** 10.4178/epih/e2014001

**Published:** 2014-04-30

A very warm welcome to you all!

I am happy to extend warm greetings to all of you as the new Editor-in-Chief. This year marks the 35th anniversary of Epidemiology and Health, the journal of the Korean Society of Epidemiology.

The Korean Society of Epidemiology was founded in 1979. In October 2009, on the society’s 30th anniversary, the Korean Journal of Epidemiology was relaunched as an open-access online journal under the new name of Epidemiology and Health (*epi*H), altering the language from Korean to English. Thanks to the devoted efforts of Professor BoYoul Choi, former Editor-in-Chief, and enthusiastic member support, our journal is currently listed in the databases of PubMed, PubMed Central, KoreaMed, Korea Journal Citation Index, CINAHAL, EBSCO host, and CABI. We have redoubled our efforts to have the journal listed in Scopus and SCI as soon as possible so that it can play a more pivotal role as an international journal.

This year, to achieve this goal, we have introduced the following seven editorial changes for *epi*H.

First, our journal is published in English, but submissions in Korean are also accepted. The submitted Korean manuscripts, after being reviewed and revised, will be translated and published.

Second, all costs, including those incurred during publication, reviewing, translation, and editing, will be assumed by the Society; authors submitting their papers are not required to assume any financial burden. Further, readers worldwide will have free access to the papers continuously.

Third, besides the existing original papers, editorial, reviews, outbreak investigation, and briefs, a variety of new sections, such as cohort profiles, data resource profiles, invited lectures, invited editorials, and book reviews, will enrich the journal.

Fourth, the primary emphases of each paper will be condensed into two to three lines, written in Korean, as key messages. This will allow Korean readers to quickly glimpse the paper’s content. Such key messages will be notified to all members by e-mail.

Fifth, all the published papers in our journal, from the inaugural issue to the latest issue, will be uploaded in full length to the *epi*H website.

Sixth, the online submission system was introduced to facilitate the submission process and enable authors to track the review process.

Finally, we launched a fast-track review to accelerate the publication of your valuable manuscripts. If you wish to profit from this fast-track review, simply let us know when submitting your manuscript.

We are currently exploring various improvement measures for the development of *epi*H, and we rely on your active participation during the process of converging productive opinions.

Our journal publishes a wide spectrum of epidemiological papers related to medicine and public health. The *epi*H editorial board will do its utmost to ensure that your valuable papers are read and cited by target audiences worldwide at the earliest possible time.

*epi*H is waiting for your esteemed manuscripts.

On behalf of the editorial board, I wish you all good health and success in all your undertakings.

Thank you. **Moran Ki** Editor-in-Chief

